# Tic Disorder of Children Analyzed and Diagnosed by Magnetic Resonance Imaging Features under Convolutional Neural Network

**DOI:** 10.1155/2021/8997105

**Published:** 2021-11-11

**Authors:** Chunxia Wu, Qingerile Si, Budegerile Su, Lan Mu, Gaowa Bao, Musiguleng Ji, Daohu Ao

**Affiliations:** Combination of Mongolian and Western Medicine with Pediatrics, Affiliated Hospital of Inner Mongolia University for The Nationalities, Tongliao 028000, Inner Mongolia Autonomous Region, China

## Abstract

This work aimed to explore the analysis and diagnosis of children with tic disorder by magnetic resonance imaging (MRI) features under convolutional neural network (CNN), to provide a certain reference basis for clinical identification. A total of 45 children diagnosed with tic disorder in hospital from January 2018 to June 2020 were selected as the research subjects. A total of 30 normal children were selected as the control group. MRI images were collected, and CNN was constructed for image processing. The results showed that the convolutional neural network could significantly improve the speed of MRI reconstruction and can improve the diagnostic accuracy. Compared with normal children, the metabolites in children with tic disorder were slightly increased, but there was no statistical significance (*P* > 0.05). The results of the Yale score showed that the proportion of children with moderate illness was significantly greater than that of children with mild and severe illness. In short, the pathological changes of tic disorder were effectively discovered by MRI based on CNN algorithms, which can provide a reference for clinical identification.

## 1. Introduction

Tic disorder is a complex and chronic neuropsychiatric disorder, which has the characteristics of high frequency, involuntary, sudden, repetitive, nonrhythmic, stereotyped, single- or multipart muscle tics, or (and) vocal tics [1]. The current research results show that genetics, neurobiochemical abnormalities, brain structure or dysfunction, and so on are common causes of this disease. The main clinical manifestations of the child are motor and vocal tics. The simple forms of motor tics are blinking, shrugging nose, crooked mouth, shrugging, turning or slanting shoulders, and so on. The twitching can occur in a single part or multiple parts of the body, and its complex forms include jumping, running, spinning, and bending behaviors [2–4]. The simple forms of vocal tics are throat clearing, roaring, sniffing, dog barking, and so on, and its complex forms are repetitive speech, imitating speech, and so on. [5]. The disease mostly occurs in children and adolescents, and it can be as high as 40% in children under five years of age. Motor tics usually occur before the child is seven years old, while vocal tics usually occur before the child is eleven years old. It is classified into three clinical types: transient tic disorder, chronic movement or vocal tic disorder, and Tourette syndrome according to the above age of onset, course of the disease, clinical manifestations, and whether it is accompanied by vocal tics [6–8]. The occurrence of the disease has a great impact on the physical and mental health of children. Therefore, early detection and timely and effective treatment are the keys to the disease [9]. At present, proton magnetic resonance spectroscopy is considered clinically as a feasible noninvasive detection method for detecting changes in brain metabolism, but there are no many relevant literature reports on the application of this method to children with tic disorder [1]. The convolutional neural network (CNN) model had good applications in image segmentation, lesion detection, image super-resolution, image noise reduction, and so on for several years [10]. The CNN model is used in medical image processing to realize the intelligentization of lesion recognition, disease classification, and diagnosis [11]. And a large number of studies have confirmed this view [12].

To improve the efficiency of clinical diagnosis of children with tic disorder and the treatment effect of children, realize the intelligence of clinical diagnosis, and reduce the work pressure of doctors, 45 children with tic disorders were selected in the research for magnetic resonance imaging (MRI) examination. Then, the image was processed by the CNN algorithm, which aimed to explore the analysis and diagnosis effect of MRI features on children with tic disorder.

## 2. Materials and Methods

### 2.1. General Materials

Forty-five children with tic disorder admitted to the hospital from January 2018 to June 2020 were selected as the research subjects, including 26 male children and 19 female children; the age range of children was 4–17 years old; and the average age was 10.54 ± 1.34 years old. There were 18 cases of Tourette's syndrome, 12 cases of chronic Tourette's disorder, and 15 cases of temporary Tourette's disorder. The shortest course of the disease was one month, and the longest time was ten years. All children obviously showed asymmetry of bilateral tic symptoms, and the child had twitches on one side of the face or limbs, or the eyeballs squinted to one side frequently. The child was examined by MRI during the onset of the disease. At the same time, 30 healthy children who received health examination in this hospital during the same period were selected as the control group, including 15 male children and 15 female children; the age of children was 5–18 years old; the average age was 9.04 ± 1.22 years old; and they were examined by MRI. The diagnostic criteria referred to the relevant diagnostic criteria in Clinical Manifestations and Diagnosis of Tic Disorders. This study had been approved by the ethics committee.

Inclusion criteria were as follows: (1) children who met the above diagnostic criteria, (2) children who were not allergic to the relevant treatment and examination drugs used in the treatment or examination in the research, and (3) children whose family members were aware of and had signed an informed consent form.

Exclusion criteria were as follows: (1) children with cognitive impairment, (2) children with poor compliance and unable to cooperate with the investigator, (3) children who had recently received relevant medications, and (4) children with congenital heart disease and so on.

### 2.2. Magnetic Resonance Detection Method

The magnetic resonance diagnostic apparatus (model: Optima 360; Siemens Avanto) was adopted to examine patients, and the body was imaged with a phased array surface coil.

The children were in the supine position, with both hands on the sides of the body, and the desired scanning range was wrapped by the body coil so that the target area was completely covered. The single point was used to resolve the spin-echo sequence. The parameters were set as follows: echo time was 1,500 ms, repetition time was 470 ms, and voxel size was 21 mm*∗*20 mm*∗*21 mm. The key areas of interest of transverse position were in the basal ganglia and the contralateral areas. The signal intensity of the main metabolites in the spectrum was automatically analyzed and measured by the software, and the metabolites mainly included N-acetyl aspartate, creatinine, and complex choline. The echo time was 130 ms, and the repetition time was 4,350 ms when the FSE/T2WI scanning was performed. The echo time was 20 ms, and the repetition time was 5,635 ms when the reverse recovery sequence scanning was performed. The echo time was 100 ms, and the repetition time was 4,475 ms when axis FSE/T2WI sequence scanning was performed. The scan layer spacing and layer thickness were both set to 5.2 mm. It took about 6–8 min to complete the regular scan. The state of the child was observed. If the state was abnormal, the scan was stopped immediately, and the appropriate treatment was given.

### 2.3. CNN Model

The main components of CNN included the data input layer, convolution layer, activation function, other processing layers, and output layer.

First of all, the input data was accepted mainly by the input layer. The input layer was usually multidimensional, and the structural information of the data itself in the picture was completely retained. The convolution kernel was used by the convolutional layer to extract features from the first input data, and the convolution kernel can usually change with the depth of the network. The basic information such as lines and contours were extracted after convolution, and this information was determined by the position of the convolutional layer in the network, that was, the deeper the convolutional layer, the larger the receptive field. Therefore, the more local features considered in fusion, the more abstract the extracted features. First, the statistics of the characteristics of the noise were carried out, and the approximate model of the noise is shown in the following equation:

(1) $$\begin{array}{c} pi=pi-1◎qi+ai, \end{array}$$

where *pi* − 1 represented the input of the l-th layer, ◎ represented the convolution operation, *qi* was the number of convolution layers, and *ai* represented the bias. Finally, the extracted feature map *pi* was the output of the first layer.

The activation function was an important part of the deep CNN, which can introduce nonlinear operations into the entire model. If there were no activation functions, other linear operation layers were superimposed to increase the depth of the network, which made the expressiveness of the whole network bad. The different activation functions got different gradient derivatives when the network propagated back. In the development of deep learning networks, the specific operations are shown in the following equations:

(2) $$\begin{array}{c} y=\frac{1}{\left(1+e-i\right)}, \end{array}$$

(3) $$\begin{array}{c} \text{tanh}\left(i\right)=\frac{\left(1-e-2i\right)}{\left(1+e-2i\right)}, \end{array}$$

(4) $$\begin{array}{c} y=\left\{\begin{array}{cc} i, & i\geq 0,\\ 0, & i＜0, \end{array}\right) \end{array}$$

where *e* is a natural number, *e*=2.718281828459045.

The pooling was used to perform local pooling operations on the feature map. This operation can remove part of the redundant information, reduced the feature map, reduced the amount of calculation, and kept the position of the feature in the image unchanged. It also helped the model prevent overfitting. The existing conventional pool operation window areas did not overlap, such as the common average pooling and maximum pooling. Overlapping pooling, as the name implies, meant that the areas of adjacent pooling operations were overlapped. There was also pyramid pooling that can convert image convolutional feature pooling of any scale into the same dimension. Each node in the fully connected layer was connected to all the nodes of the previous layer, and the features extracted from the previous layer were synthesized and output to the output layer. Generally, the parameters of the fully connected layer were the most due to the connected characteristics of the fully connected layer. The entire training process was passing the input first; after the forward propagation had gone through all levels and processing, the loss of this round was calculated according to the loss function; and then the backward propagation loss was calculated. The appropriate optimization method, such as stochastic gradient descent, was used to update the parameters of each layer in the direction of reducing the loss.

A T-layer CNN *Y*=*L*(*x*, *o*) was assumed to be expressed as follows:

(5) $$\begin{array}{c} \left\{\begin{array}{c} L_{0}=X,\\ L_{l}=\Phi_{l}\left(\Omega_{l}*L_{l-1}+b_{l}\right),\;l=1,2,\ldots ,L-1,\\ L_{T}=\Omega_{T}*L_{T-1}+b_{L}, \end{array}\right) \end{array}$$

where *L*_0_ represented the input of the CNN, Ω_*l*_ represented the convolution operation, *b*_*l*_ represented the vector bias, and Φ_*l*_ was used for nonlinear activation.

In addition to forward propagation, back propagation can be applied to the calculation of reverse gradients. As a result, the parameters of the network were updated, which was also an important process of the CNN training segment. The training set (*x*, *y*) was given, and then the minimized loss function was established, as shown in the following equation:

(6) $$\begin{array}{c} \theta =\arg\;\min L\left(x,y\right). \end{array}$$

Therefore, the loss function had a great influence on the quality of the final learning result.

The above model was applied in the MRI results of the research. Because the model was affected by the operation, time, environment, and instruments during the imaging process, the results showed that the specifications were not uniform, the illumination was uneven, and the quality was different. To reduce the interference of these factors in subsequent feature extraction, it was necessary to perform some preprocessing on the image results.

First, the binarization threshold was used for segmentation to denoise. The imaging image needed to be converted to a grayscale image to facilitate the processing of digital image algorithms. Then, the between-class variance method was selected for binarization threshold segmentation. The specific operation was that the target of the image was separated from the background; the size of the image *P*(*m∗n*) was set to *m∗n*; and the initial threshold was *L*. The number of pixels in the pixel set *T*_1_ whose gray level was lower than the threshold was recorded as *I*_1_, and the number of pixels in the pixel set *T*_2_ that was higher than the threshold was recorded as *I*_2_. The condition that needed to be met was *I*_1_+*I*_2_=*a∗b*. Meanwhile, the proportions of *I*_1_ pixels and *I*_2_ pixels were established, as shown in the following equations:

(7) $$\begin{array}{c} Q_{1}=\frac{I_{1}}{m*n}, \end{array}$$

(8) $$\begin{array}{c} Q_{2}=\frac{I_{2}}{m*n}, \end{array}$$

(9) $$\begin{array}{c} Q_{1}+Q_{2}=1. \end{array}$$

The average gray *Y* corresponding to these two parts was established, as shown in the following equations:

(10) $$\begin{array}{c} Y_{1}=\frac{\sum_{\left(a,e\right)\in Y_{1}}Y\left(a,e\right)}{I_{1}}, \end{array}$$

(11) $$\begin{array}{c} Y_{2}=\frac{\sum_{\left(a,e\right)\in Y_{2}}Y\left(a,e\right)}{I_{2}}, \end{array}$$

where *Y*(*a*, *e*) was the pixel value. The average gray value of the entire image was established, as shown in the following equation:

(12) $$\begin{array}{c} Y=Y_{1}*Q_{1}+Y_{2}*Q_{2}. \end{array}$$

The gray value variance between the two types of pixels was presented, as shown in the following equation:

(13) $$\begin{array}{c} \Delta \left(T_{1},T_{2}\right)=Q_{1}*Q_{2}*\left(Y_{1}-Y_{2}\right). \end{array}$$

The corrosion operation was used to remove the surrounding noise blocks, and the final binary image was obtained.

The illumination of the image was not uniform, so lighting needed to be processed, the overall image brightness needed to be balanced, and the available data of the model needed to be increased to reduce the interference of subsequent feature extraction. The image's illuminance-reflection model was established as shown in the following equation:

(14) $$\begin{array}{c} W\left(i,l\right)=G\left(i,l\right)*K\left(i,l\right), \end{array}$$

where *G*(*i*, *l*) and *K*(*i*, *l*) represented the light component and the reflected component, respectively, which can reflect the low- and high-frequency characteristics of the image. If the illumination was found to be uneven, it indicated that the spatial distribution of the *G* component in the picture was uneven. Therefore, the *G* component needed to be corrected. The multiscale Gaussian function and the luminance component *W*(*i*, *l*) of the original spatial image needed to be used for convolution to extract the illumination component in the image, as shown in the following equation:

(15) $$\begin{array}{c} Z\left(i,l\right)=W\left(i,l\right)*E\left(i,l\right), \end{array}$$

where *E*(*i*, *l*) represented the illumination component of the image and *Z*(*i*, *l*) represented the selected Gaussian function, as shown in the following equation:

(16) $$\begin{array}{c} Z\left(i,l\right)=\lambda \;\exp\left(-\frac{i^{2}+l^{2}}{q^{2}}\right), \end{array}$$

where *λ* represented the normalization constant and *q* represented the scale factor, which can determine the size of the convolution kernel of the Gaussian function. When the convolution kernel of the Gaussian function was large, partial global characteristics were extracted, and when the convolution kernel of the Gaussian function was small, local characteristics were extracted. Therefore, it was necessary to use the unused *q* for synthesis.

Then, the characteristics of the light distribution were used to adjust, and then the gamma transform was used to correct, as shown in the following equation:

(17) $$\begin{array}{c} U\left(i,l\right)=255{\left(\frac{W\left(i,l\right)}{255}\right)}^{\gamma }, \end{array}$$

where *U*(*i*, *l*) represented the output value, and the enhancement index of brightness is shown in the following equation:

(18) $$\begin{array}{c} \gamma =0.5^{1-\left(E\left(i,l\right)/n\right)}, \end{array}$$

where *n* represented the mean value of the care component *E*.

### 2.4. Statistical Analysis

The collected data was sorted and summarized, and SPSS 23.0 was used for statistical analysis. The measurement data were expressed as mean ± standard deviation $\left(\bar{X}\pm s\right)$. The single sample data were tested by *t* test. The count data were tested by *x*^2^ test, which were expressed as case number (%), and *P* < 0.05 indicated that the difference was statistically remarkable.

## 3. Results

### 3.1. CNN Model Testing

**Figure 1:**
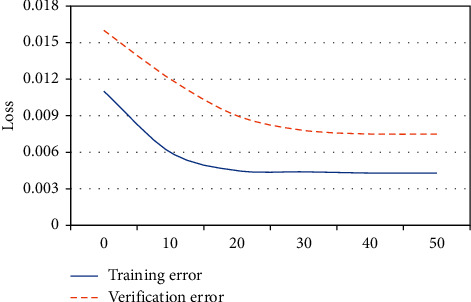
CNN model testing.

Figure 1 shows that, with the number of iterations increased, both the training error and the verification error gradually tended to converge, and the verification error was slightly higher than the training error. This showed that the model training was relatively reasonable.

### 3.2. Analysis of Reconstruction Time of CNN Model

**Figure 2:**
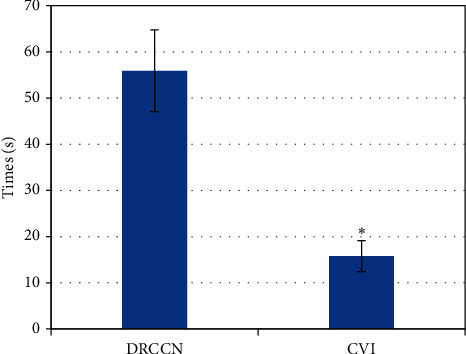
Analysis of reconstruction time of CNN model (^∗^*P* < 0.05).

Figure 2 shows that the reconstruction time of the CNN was much faster than the traditional parallel imaging method, and the difference was statistically significant (*P* < 0.05).

### 3.3. Image Inspection Results

**Figure 3:**
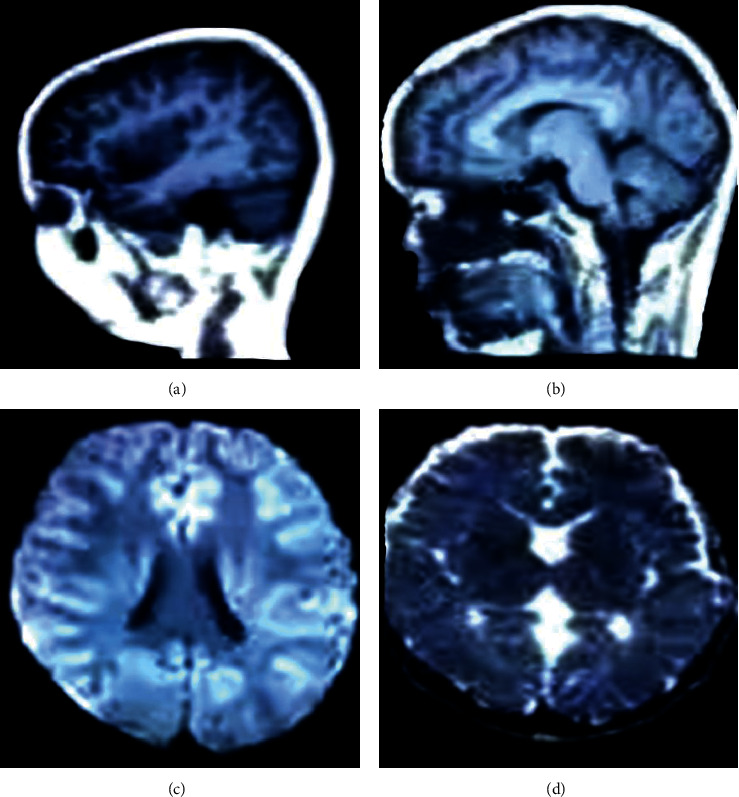
MRI results: (a) lateral ventricle, (b) cerebral aqueduct, (c) fourth ventricle, and (d) third ventricle.

**Figure 4:**
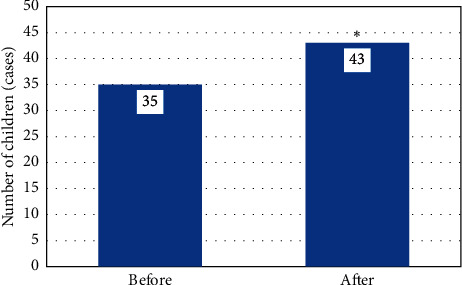
Imaging diagnosis rates before and after the algorithm was used (^∗^*P* < 0.05).

**Figure 5:**
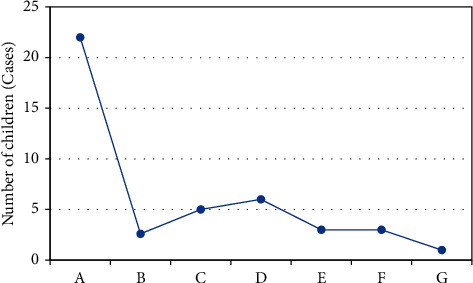
Imaging test result. A: healthy child; B: widening of intracranial space; C: abnormal signal focus; D: widening of the ventricle; E: intracranial cyst; F: lacuna focal; G: hemangioma.

In Figure 3, the MRI examination showed that the lateral ventricle of the ventricle was significantly enlarged, and there was a clear perivascular space, accompanied by the cribriform signs of the deep white matter around the ventricle. The hemangiomas were faintly visible, and abnormal signal foci were prominent.  Figure 4 shows the diagnosis rate of MRI. It was found that the accuracy of MRI diagnosis after using the algorithm was significantly improved, and the difference was statistically substantial (*P* < 0.05).  Figure 5 shows the results of the examination. It was found that the majority of children had no pathological abnormalities in the brain. There were many children with ventricular widening in abnormal lesions, and the least children with hemangioma.

### 3.4. Metabolites Test Result

**Figure 6:**
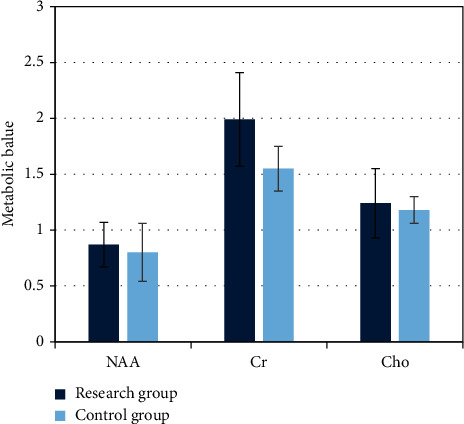
Metabolites test result. NAA: n-acetylaspartic acid; Cr: creatinine; Cho: choline.

Figure 6 shows that the metabolites of the children in the study group were higher compared with the control group, but the difference was small, and it was not statistically significant (*P* < 0.05).

### 3.5. Yale Score Test Result of Children

**Figure 7:**
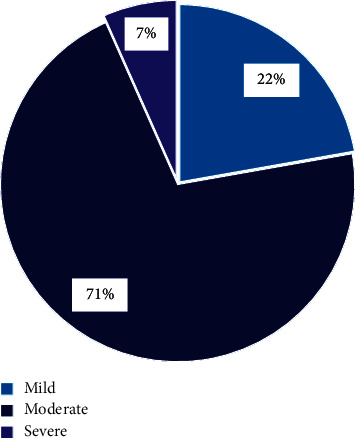
Yale score test result of children.

The children were classified into three groups: mild children (0–24 points), moderate children (25–49 points), and severe children (>50 points) according to the Yale score. Among them, the proportion of children with moderate illness was the highest, as shown in Figure 7.

### 3.6. Abnormal Rate of Magnetic Resonance in Children with Tic Disorder of Different Degrees

**Figure 8:**
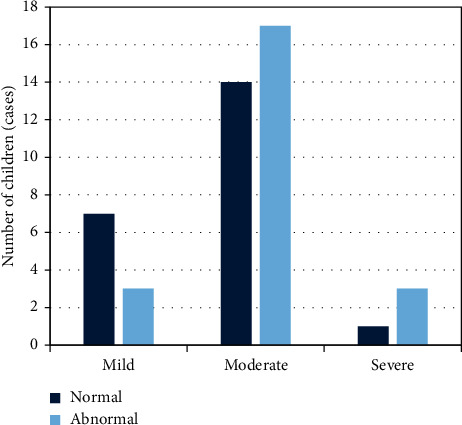
Abnormal rates of magnetic resonance in children with tic disorder of different degrees.

Figure 8 shows that the higher the Yale score, the more children with abnormal magnetic resonance examinations.

## 4. Discussion

The pathogenesis of tic disorder has not yet been fully clarified, and previous studies have involved many fields such as neurobiochemical and neuroimaging. However, the research on the neuroimaging of the disease mainly uses EEG examinations, and there are few related literatures on magnetic resonance examinations [13–15]. In recent years, it has provided many evidences for the etiology and diagnostics of tic disorders with the continuous optimization of magnetic resonance examination technology [16]. Magnetic resonance examination was the main definitive examination method for the diagnosis and differential diagnosis of central nervous system diseases [17].

The early detection of tic disorder was particularly important, which can effectively avoid adverse effects on the healthy growth of children in the future [18–20]. In the research, the magnetic resonance examination method based on the CNN algorithm was used. The result showed that with the number of iterations increased, the training error and the verification error gradually tended to converge, and the verification error was slightly higher than the training error. This showed that the model training was relatively reasonable. Moreover, the reconstruction time of the CNN was much faster than the traditional parallel imaging method. It showed that the accuracy of imaging was improved by this algorithm and the processing time was saved, which was beneficial for researchers to obtain objective and true imaging results in a short time. This is consistent with the result that Li et al. applied the CNN model to the segmentation of prostate MRI images, which can shorten the labeling time and have a relatively high standard accuracy [21]. Magnetic resonance examination found that the lateral ventricles of the ventricles of some children were significantly enlarged, there were obvious perivascular spaces, accompanied by cribriform signs of deep white matter around the ventricles, hemangioma was faintly visible, and the abnormal signal foci were prominent. However, there were also children with no pathological abnormalities, many children with ventricular widening in abnormal lesions, and the least children with hemangioma. A special sequence of magnetic resonance was used to examine the metabolites of the brain, and it was found that the metabolites of the children in the study group were high. It was not statistically significant because the difference was small. In addition, the children were also scored by Yale in the research, and the children after the scoring were classified into groups. It was found that the higher the Yale score, the more children with abnormal MRI compared with the results of the magnetic resonance examination. It showed that the results of magnetic resonance examination had a high reference value.

## 5. Conclusion

In order to improve the efficiency of clinical diagnosis of children with tic disorder, it first established a convolutional neural network algorithm model and applied it to the results of magnetic resonance imaging. As a result, the diagnosis time could be shortened compared with the traditional method, and the diagnosis accuracy could be ensured. However, this study still has certain limitations. For example, this study does not fully reflect the results of magnetic resonance imaging, so it will be further studied in future work. In summary, magnetic resonance imaging based on the convolutional neural network algorithm can effectively find the pathological changes of tic disorders and provide a reference for clinical identification.

## Data Availability

No data were used to support this study.
